# Editorial to “Mid‐term comparison of new‐onset AHRE between His bundle and left bundle branch area pacing in patients with AV block”

**DOI:** 10.1002/joa3.70029

**Published:** 2025-02-24

**Authors:** Takashi Noda

**Affiliations:** ^1^ Department of Cardiovascular Center Kindai University Osaka‐Sayama Japan

Pacemaker implantation with right ventricular pacing is widely used in clinical practice in the treatment of bradycardia, especially in patients with symptomatic AV block. However, right ventricular apical pacing (RVAP) sometimes induces electromechanical dyssynchrony, leading to adverse clinical impacts on clinical outcomes, including an increased risk of new‐onset atrial arrhythmias. Physiological conduction system pacing (CSP), His bundle pacing (HBP), and left bundle area pacing (LBBAP) are recommended for patients with reduced left ventricular (LV) systolic function and substantial ventricular pacing (>20%) since CSP has been reported to improve clinical outcomes compared with RVAP. Although several studies suggest that CSP is associated with a lower incidence of new‐onset atrial arrhythmias detected as atrial high‐rate episodes (AHRE), the performance between HBP pacing and LBBAP on the risk of new‐onset AHRE remains unclear.

Pestrea et al. showed that HBP and LBBAP were associated with a similar incidence of device‐detected new‐onset AHRE during a medium‐term follow‐up period in patients with atrioventricular block.^1^ They compared the incidence of device‐detected new‐onset AHRE between the two groups of patients after HBP (*n* = 59) and those after LBBAP (*n* = 83) during a mean follow‐up of 624 days. New‐onset AHRE occurred in 8 (13.5%) in the HBP group and in 14 (16.8%) in the LBBAP group. Multivariable Cox regression analysis showed that HBP and LBBAP had similar predictive values for device‐detected new‐onset AHRE. Moreover, there was no significant difference between the two groups regarding the total burden of AHRE, which was less than 1% in almost all patients with new‐onset AHRE, although there were several limitations such as using different criteria of the current 2020 ESC guideline, which indicated the device‐programmed rate criterion for AHRE is greater than or equal to 175 bpm and the duration criterion is greater than or equal to 5 min.^2^

Cardiac electronic implantable devices such as pacemakers have the ability to monitor rhythm abnormalities, which allow us to recognize a new entity of AHRE easily. From a clinical point of view, AHRE has been associated with the development of clinical atrial fibrillation (AF) and an increase in stroke and death risk. There have been reports about the risk factors for AHRE including older age, left atrial volume, prior history of AF, white cell count, high levels of C reactive protein, and CHADS2 score.^3^ As for the issue related to new‐onset AHRE after implantation, a high burden of RVAP is a risk for increased AHRE since RVAP induces paradoxical septal motion and ventricular dyssynchrony. As a result, increased filling pressure in each heart chamber leads to electric remodeling of the left atrium. CSP restores ventricular contraction synchrony by pacing the His‐Purkinje conduction system directly, which allows for rapid and widespread dissemination of ventricular activation throughout the ventricle. CSP, including both HBP and LBBAP, has several advantages of LV function, subsequent events of heart failure hospitalization, and the incidence of new‐onset AHRE during a follow‐up compared to RVAP, especially in patients with LV dysfunction and a high burden of right ventricular pacing.^4^

There are some limitations of relatively low procedural success rates and the development of a high and unstable pacing threshold in terms of HBP, although it has demonstrated several clinical benefits. In addition, HBP is inefficient as physiological pacing if a patient has infra‐Hisian distal conduction block. There are several strong points of LBBAP, including the wide target area of left bundle and Purkinje fibers on the LV septum and a stable low pacing threshold with no significant sensing issues. In fact, a previous meta‐analysis revealed that LBBP was significantly associated with higher implant success rates (relative risk: 1.12), lower capture threshold at implantation (mean difference [MD]: 0.63 V at 0.5 ms) and lower capture threshold at follow‐up (MD: 0.76 V at 0.5 ms) compared with HBP.^5^ These data suggest that pacing characteristics are better in LBBAP than in HBP; however, the incidence of new‐onset AHRE during a follow‐up may be similar between patients with LBBAP and those with HBP by taking the current issue into consideration. At this moment, it remains controversial which we should select: LBBAP or HBP. Large‐scale, randomized control studies are warranted to reveal the true answer.

## CONFLICT OF INTEREST STATEMENT

Dr. Noda reports Grants‐in‐Aid for Scientific Research (22K08092) from the Ministry of Education, Culture, Sports, Science, and Technology of Japan and declares receiving fees for speakers from Medtronic Japan and Biotronik Japan.
